# Variations in the patterns of tobacco usage among indian females - findings from the global adult tobacco survey India

**DOI:** 10.1186/s12905-022-02014-3

**Published:** 2022-11-11

**Authors:** Lajya Devi Goyal, Madhur Verma, Priyanka Garg, Garima Bhatt

**Affiliations:** 1 Department of Obstetrics and Gynecology, All India Institute of Medical Sciences, 151001 Bathinda, Punjab India; 2 Department of Community and Family Medicine, All India Institute of Medical Sciences, 151001 Bathinda, Punjab India; 3 Department of Community Medicine, School of Public Health, Post Graduate Institute of Medical Education and Research (PGIMER), Chandigarh, India

**Keywords:** Women’s health, Tobacco usage, Smokeless tobacco, Pack health warnings

## Abstract

**Background:**

Males dominate in tobacco usage, as well as in tobacco research, knowing that women face more severe health consequences. There is a specific lack of information on epidemiological statistics, risks, and the level of knowledge among women regarding tobacco. This study examines the Global Adult Tobacco Survey (GATS)-India dataset to estimate female tobacco usage and assess socio-economic variations in tobacco consumption, awareness regarding the adverse effects of tobacco, noticing pack health warnings (PHW), and intention to quit tobacco use well as factors influencing these domains.

**Methods:**

Using a geographically clustered multistage sampling method, the nationally representative GATS II (2016–17) interviewed 40,265 female respondents aged 15 years and above from all Indian states and union territories. Standard operational definitions were used to estimate the primary independent variables (community, individual, and household categories) and dependent variables like awareness regarding the adverse effects of tobacco, noticing pack health warning (PHW), and intention to quit tobacco. Sampling weights were adjusted while performing the analysis. Bivariate and multivariable analysis were used to generate the estimates.

**Results:**

Of the total female respondents, 84.2% were never-users, 13.3% ever consumed Smokeless Tobacco (SLT) products, 1.8% ever smoked tobacco, and 0.8% were dual users once in their lives. Around 16% of the women had exposure to Second Hand Smoke (SHS) either at their homes, workplaces or in public places. Overall, maximum awareness was seen among non-smoker females (64.7%) and dual users (64.7%), followed by women exposed to SHS, SLT users, and smokers. PHW was noticed more by the bidi smokers, followed by SLT users and cigarette smokers. Factors that positively affected intention to quit smoking included younger age, secondary school education, self-employed status, the habit of buying packed cigarettes/bidi, believing that smoking causes serious illness, and attempted quitting in the last 12 months.

**Conclusion:**

A high proportion of women consume tobacco which is significantly influenced by socio-demographic factors. Tobacco regulators should be especially concerned about women as the tobacco marketing experts target them. Mobilizing self-help groups and organizations working for women and children could assist broader campaigns to generate awareness and motivate quitting attempts.

## Introduction

Overall global tobacco use has decreased over the last two decades, from 1.397 billion in 2000 to 1.337 billion users in 2018. The age-standardized tobacco use prevalence rates are also declining in all World Health Organization (WHO) regions. [1]. The tobacco industry foresaw the declining trend in its very initial stages. Hence, they started shifting their targets to find newer business avenues in the Low and Middle-Income Countries (LMIC), making them more vulnerable to the tobacco epidemic. [2–5]. The exaggerated efforts by the tobacco industries increased the participation and indulgence of both men and women from LMICs in consuming tobacco [6]. Consequently, a state of an epidemic of tobacco-related diseases has been created in these countries, where tobacco usage is rapidly becoming a pertinent public health issue. Furthermore, the WHO Southeast Asian Region has the highest tobacco consumption rates, with an estimated (2018) 29.1% of adults aged 15 years and older using tobacco in any form. [1, 7, 8].

Until 2016, India was the world’s 2nd largest tobacco consumer, trailing only China [9]. Similar to global patterns, the tobacco epidemic in India is gradually declining. The most recent round of the Global Adult Tobacco Survey (GATS) conducted between 2016 and 17 showed a 6% decrease in tobacco use in Indian adults(> 15 years). Compared with the previous round, the overall tobacco usage in India has decreased relatively by around 11.5% and 30% in males and females, respectively [10]. However, the preliminary reports from the fifth round of the National Family Health Survey (NFHS-5) (2019-20) also confirm the declining trends and corroborate with the GATS-2 to depict that the declining trends are not seen all over the country [11]. Tobacco usage has decreased among men in most Indian states, except Sikkim, Goa, Bihar, Gujarat, Himachal Pradesh, and Mizoram. The prevalence of women’s tobacco consumption has also decreased in all states except Mizoram and Sikkim [11]. These disparities suggest that tobacco control programs require more focused interventions for vulnerable groups of people. Furthermore, the emphasis on men portrays gender bias and the inequality that underpins many tobacco control programs [12, 13]. Over the last few decades, policymakers and implementers have become increasingly concerned about the alarming rise in tobacco use among women in developed and developing nations [14]. This is because an increase in the number of female tobacco users will have a significant negative impact on household finances and family health [15].

Tobacco exerts strong adverse effects on women’s health due to premature menopause attributed to its anti-estrogen effect. While tobacco reduces the risk of endometrial cancer as per Felix et al. (2014) [16]. , it increases the risk for premature menopause, which in turn enhances the risk of cardiovascular disease and osteoporotic fractures [17]. Also, tobacco usage is causative of several gynecological problems, including cancers. There is a direct association between tobacco use in reproductive age groups and breast cancer, especially if smoking begins while the woman is nulliparous [18]. Tobacco exacerbates cervical intraepithelial neoplasia and has been related to cervical squamous cell carcinoma in women seropositive for the Human Papillomavirus16 and 18 [19].

The high percentage of non-smoking women makes them an attractive target for the tobacco industry. Their efforts to promote tobacco usage are supported by the dearth of adequate awareness regarding the adverse effects of tobacco, prevalent myths around smoke, and SLT products. Unless sustained and efficient measures are implemented, the prevalence of female tobacco use is expected to rise. Article 4 of the World Health Organization- Framework Convention on Tobacco Control (WHO-FCTC) Guiding Principles raised concerns about gender disparity in tobacco control efforts. It emphasized the “need for taking measures to address gender-specific risks when developing tobacco control strategies.”[20].

There is a specific lack of information on epidemiological statistics, risks, and the level of knowledge among women regarding tobacco. Robust evidence will effectively guide tobacco control policies, resulting in substantial gains in public health and reduced morbidity and mortality, especially when viewed through a gender parity lens. Tobacco usage trends, in this regard, are an essential source of insight at the national and sub-national levels for monitoring the effectiveness of existing policy initiatives and determining future directions. Over the last decade, GATS-India has assessed tobacco prevalence and pattern of use at various points in time. This secondary data analysis attempts to review GATS datasets to estimate the prevalence of tobacco use among females. The study’s specific objectives were to comprehensively understand the socio-economic variations in tobacco usage, their awareness of the harmful effects of tobacco, noticing the PHW, their intention to quit tobacco usage, and factors influencing these domains.

## Methodology

### Source of data

We used data from the second wave of GATS-India (2016-17) [9]. It is a cross-sectional national survey conducted by the Tata Institute of Social Sciences designated by MoHFW, Government of India. GATS-2 included all states of India. This survey followed a standard protocol for the study design, data collection, and study tool development. GATS-2 addressed tobacco usage (smoking and SLT), SHS exposure, cessation, economics, media messages, knowledge, attitude, and perceptions of tobacco use.

### Sample selection

**Fig. 1:**
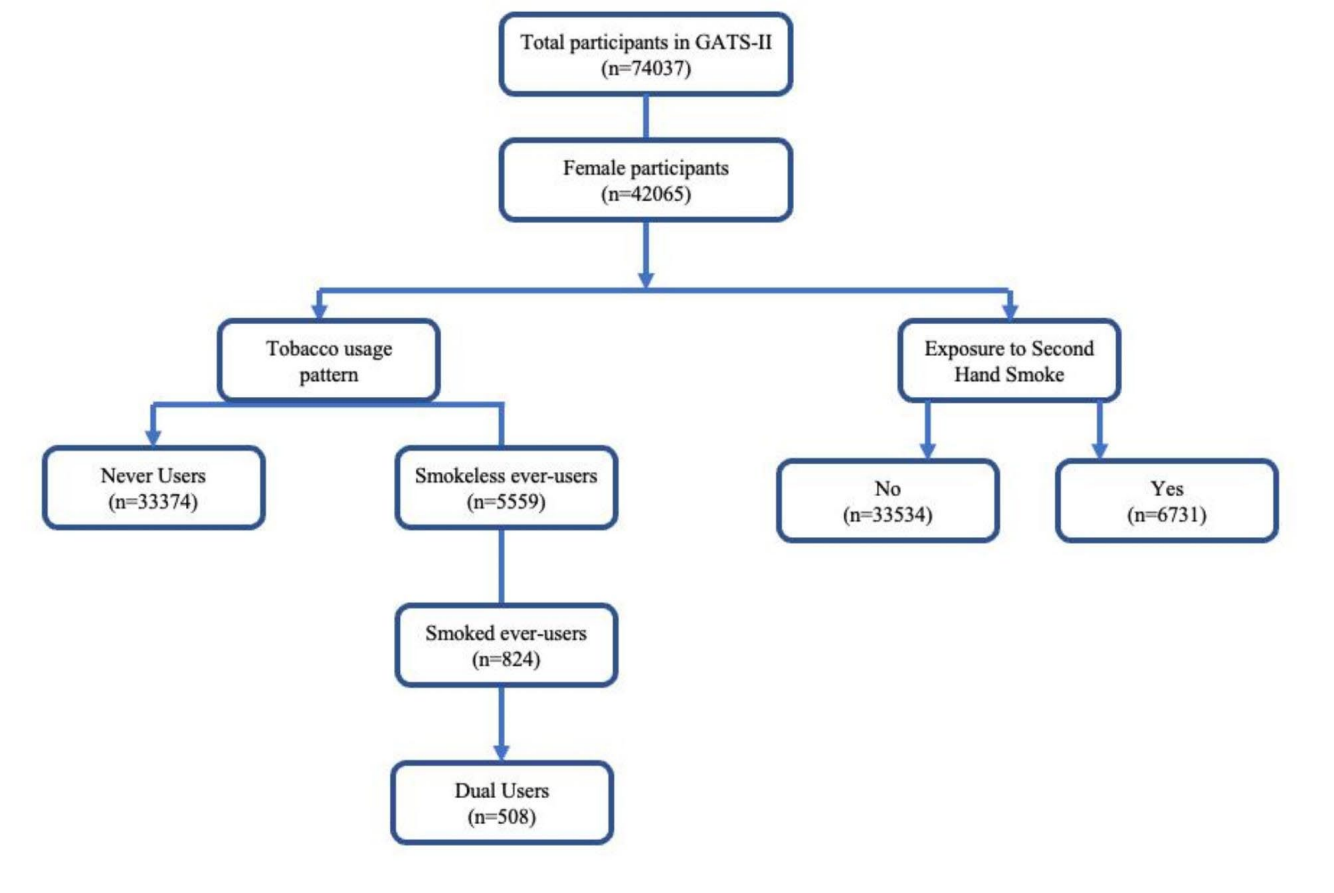
Sample selection flowchart

In total, 40,265 female respondents (≥ 15 years) were included in the GATS-2 final sample **(**Fig. 1**).** The Operational definitions of the tobacco consumption variables used in the analysis like ‘Ever tobacco users,’ ‘Smoking tobacco (ever) users,’ ‘SLT users (ever users),’‘ Dual users,’ and ‘Never-users’ were defined as per the standard GATS methodology [21]. Further, ‘Second-hand smoking’ was determined based on our previous methods. [22].

## Study variables

### Dependent variables

- **Comprehensive awareness about the adverse effects of tobacco**: Comprehensive awareness of the respondent was defined as the respondent being aware of all of the severe illnesses caused by tobacco including stroke, heart attack, lung cancer, chronic cough/Tuberculosis, and SLT causes any severe illnesses or oral cancer, dental diseases or that SLT during pregnancy causes harm to the fetus. The details about this variable are described in detail elsewhere [23].
- **Noticed PHW**: Outcome variables for noticing PHW and thought of quitting because of PHW were defined separately for smokers, bidi smokers, and SLT users. The variable was derived using the previously published methodology [24].
- **Intention to quit/Thought of quitting tobacco usage**: The variable “thinking to quit smoking because of PHW” among respondents who consumed smoking tobacco or SLT was derived from previously published literature [24].

### Independent variables

A variety of factors influence tobacco consumption. Independent variables were selected following a rigorous literature review, and the variables that significantly affected the dependent variables were included in the study. These variables were classified at community, household, and individual levels.

Regions and residence (urban/rural) were variables included in the community category. India was divided into six regions based on geographical location and cultural factors per the GATS India protocols: North, Central, West, South, East, and North East.

The household category variables included wealth index quintiles, caste (Scheduled Castes, Scheduled Tribes, Other Backward Classes, and Others), and religion (Hindu, Muslim, and Others). The variable “wealth quintile” was created using a summative score of inverse weighted proportions of possession of the following assets: electricity; refrigerator; washing machine; air conditioner; electric fan; internet connection; computer/laptop; fixed telephone; cell phone; and radio. The summative score was then divided into quintiles to obtain wealth categories (lowest, lower, middle, higher, and highest quintiles), used as a proxy for wealth or socio-economic status [25, 26].

Individual variables included age in completed years (15–24/25–44/45–64 and 65 and above), sex (male/female), level of education (no education, primary, secondary, and higher), type of occupation (government and non-government, self-employed, student, homemaker, and retired/unemployed), currently pregnant and awareness among the respondents.

### Statistical analysis

We used STATA 13 and Statistical Package for the Social Sciences (SPSS) version 25.0 to calculate our estimates, and it was represented as a weighted percentage with a 95% confidence interval. To estimate the relationship between tobacco usage (smoked, smokeless, and dual usage), socio-demographic characteristics, awareness about the harmful effects of tobacco use, noticing the PHW, and intentions to quit tobacco, the chi-square test was used to examine the associations. The unadjusted odds ratio was calculated using univariate logistic regression. Independent variables with p-values < 0.02 were considered for the multivariable logistic regression model, and the backward likelihood ratio method was used to determine the best fit model. Models were created using the complex sample analysis technique after applying sampling weights and adjusting for multistage sampling designs. Statistical significance was defined as a p-value of < 0.05.

## Results

A total of 40,625 women ≥ 15 years of age responded during the GATS-2. Amongst them, 84.2%were never-users, 13.3% ever consumed SLT products, 1.8% ever smoked tobacco, while 0.8% were dual users once in their lives. About 16.6% of the women were exposed to SHS in their homes, workplace, or public places.

Table 1 depicts the variations in the prevalence of tobacco usage amongst women as per the various socio-demographic indicators. There was an increase in the prevalence of tobacco usage with age, and it was highest in separated/divorced/widowed women from rural areas. SLT and dual usage were highest among uneducated women, and smoked tobacco was highest among women educated up to secondary school. Women from the North-eastern part of the country had the highest amount of SLT and dual tobacco users, while women from north India preferred smoking over other types of usage. We observed that women from the poorest sections of the society (first quintile) had the highest prevalence of SLT consumption, while the fourth quintile showed maximum usage of smoked tobacco or were dual users. Prevalence of SLT, smoked and dual use of tobacco was also higher amongst the women and low levels of awareness. The exposure to the SHS at any place was significantly more amongst the youngest age groups, unmarried, educated women from urban areas of North India, who were from the richest sections of society. Pregnant women and women with high awareness showed more exposure to SHS.

**Table 1:** Socio-demographic variations in tobacco usage among the female respondents as per GATS – India Round 2 (n = 40,265)

Variables	Sample Size (Unweighted counts)	Tobacco usage pattern					Second-hand smoke exposure		
		**Never users**	**SLT ever user**	**Smoked ever user**	**Dual ever user**	**p-value**	**Absent**	**Present**	**p-value**
		*Weighted %* *(95% CI)*	*Weighted %* *(95% CI)*	*Weighted %* *(95% CI)*	*Weighted % (95% CI)*		*Weighted %* *(95% CI)*	*Weighted %* *(95% CI)*	
**Total Female** **(weighted %)**	40,265	33,374 (84.2)	5559 (13.3)	824 (1.8)	508 (0.8)		33,534 (83.4)	6731 (16.6)	
**Age group (years)**									
15–29	12,424	94.3 (93.7–94.9)	5.2 (4.7–5.8)	0.3 (0.2–0.5)	0.2 (0.1–0.3)	< 0.001	82.4 (81.3–83.5)	17.6 (16.5–18.7)	< 0.001
30–44	14,276	85.2 (84.1–86.2)	13.0 (12.1–14.0)	1.2 (0.9–1.5)	0.6 (0.4–0.9)		82.4 (81.4–83.4)	17.6 (16.6–18.6)	
45– 59	8332	75.8 (74.4–77.2)	20.2 (18.9–21.5)	2.9 (2.4–3.5)	1.1 (0.8–1.6)		83.3 (82.0-84.5)	16.7 (15.5–18.0)	
≥ 60	5233	66.0 (63.9–68.1)	26.0 (24.1–28.0)	5.4 (4.6–6.4)	2.5 (1.7–3.5)		88.4 (87.1–89.6)	11.6 (10.4–12.9)	
**Marital status**									
Unmarried	4927	96.9 (96.1–97.5)	2.8 (2.2–3.4)	0.3 (0.1–0.8)	0.1 (0.0-0.3)	< 0.001	80.9 (79.1–82.7)	19.1 (17.3–20.9)	< 0.001
Married	30,994	84.3 (83.6–84.9)	13.3 (12.7–14.0)	1.7 (1.5–1.9)	0.7 (0.6-1.0)		83.5 (82.8–84.1)	16.5 (15.9–17.2)	
Separated/divorced/widowed	4340	65.4 (63.2–67.5)	27.9 (25.9–30.1)	4.5 (3.7–5.5)	2.1 (1.6–2.9)		86.7 (85.2–88.1)	13.3 (11.9–14.8)	
**Level of education**									
No formal school	13,131	71.3 (70.1–72.4)	22.9 (21.8–23.9)	4.1 (3.6–4.6)	1.8 (1.4–2.3)	< 0.001	85.6 (84.7–86.4)	14.4 (13.6–15.3)	< 0.001
Up to primary school	8300	84.0 (82.6–85.2)	14.6 (13.4–15.9)	0.9 (0.6–1.3)	0.5 (0.3–0.9)		84.6 (83.4–85.8)	15.4 (14.2–16.6)	
Up to secondary school	10,962	92.7 (91.8–93.5)	0.3 (0.1–0.5)	6.9 (6.1–7.7)	0.2 (0.1–0.3)		82.8 (81.6–83.9)	17.2 (16.1–18.4)	
Higher secondary and above	7833	97.8 (97.1–98.4)	1.9 (1.4–2.6)	0.2 (0.1–0.8)	0		79.0 (77.3–80.6)	21.0 (19.4–22.7)	
**Residence**									
Urban	14,675	89.8 (88.8–90.7)	9.3 (8.5–10.3)	0.6 (0.4–0.8)	0.3 (0.2–0.5)	< 0.001	81.6 (80.5–82.7)	18.4 (17.3–19.5)	< 0.001
Rural	25,590	81.2 (80.5–82.0)	15.3 (14.7–16.0)	2.4 (2.1–2.7)	1.1 (0.8–1.3)		84.4 (83.7–85.0)	15.6 (15.0-16.3)	
**Region**									
North	10,321	94.9 (94.2–95.5)	1.6 (1.2-2.0)	3.0 (2.6–3.5)	0.5 (0.3–0.7)	< 0.001	75.9 (74.7–77.1)	24.1 (22.9–25.3)	< 0.001
Central	5840	81.0 (79.6–82.2)	15.3 (14.2–16.5)	2.6 (2.1–3.2)	1.1 (0.8–1.7)		83.9 (82.6–85.1)	16.1 (14.9–17.4)	
East	5113	82.3 (81.0-83.4)	15.6 (14.5–16.7)	1.7 (1.3–2.1)	0.5 (0.3–0.7)		83.6 (82.3–84.8)	16.4 (15.2–17.7)	
North East	6863	61.2 (59.2–63.2)	34.7 (32.8–36.7)	1.7 (1.3–2.2)	2.3 (1.9–2.9)		85.6 (84.2–86.9)	14.4 (13.1–15.8)	
West	4281	84.1 (82.3–85.7)	14.5 (12.9–16.3)	0.5 (0.3–1.1)	0.9 (0.5–1.5)		83.3 (81.5–84.9)	16.7 (15.1–18.5)	
South	7847	89.9 (88.9–90.8)	8.4 (7.6–9.4)	1.1 (0.8–1.5)	0.5 (0.4–0.8)		85.2 (84.1–86.3)	14.8 (13.7–15.9)	
**Wealth-index Quintiles**									
First	6035	82.0 (80.4–83.4)	15.4 (14.1–16.8)	1.6 (1.2–2.1)	1.1 (0.6–1.9)	< 0.001	84.2 (82.8–85.5)	15.8 (14.5–17.2)	< 0.001
Second	7777	83.3 (82.0-84.5)	14.2 (13.1–15.4)	1.8 (1.4–2.3)	0.6 (0.4-1.0)		83.5 (82.2–84.7)	16.5 (15.3–17.8)	
Third	7819	84.1 (82.8–85.3)	13.5 (12.4–14.7)	1.8 (1.4–2.3)	0.6 (0.4–0.9)		85.7 (84.4–86.8)	14.3 (13.2–15.6)	
Fourth	8135	82.4 (81.0-83.8)	14.4 (13.1–15.7)	2.0 (1.6–2.5)	1.2 (0.8–1.7)		82.7 (81.4–84.0)	17.3 (16.0-18.6)	
Fifth	9866	90.7 (89.6–91.7)	7.8 (6.8–8.8)	1.2 (0.9–1.7)	0.3 (0.2–0.5)		80.2 (78.7–81.7)	19.8 (18.3–21.3)	
**Currently pregnant**									
Yes	6191	91.4 (88.6–93.6)	7.7 (5.7–10.3)	0.3 (0.1–1.1)	0.6 (0.1-3.0)	0.016	80.4 (76.5–83.8)	19.6 (16.2–23.5)	< 0.001
No	26,383	88.7 (88.1–89.3)	10.0 (9.5–10.6)	0.9 (0.7–1.1)	0.4 (0.3–0.5)		82.5 (81.8–83.2)	17.5 (16.8–18.2)	
Awareness*									
Unaware	18,402	80.3 (79.4–81.2)	16.1 (15.3–16.9)	2.4 (2.1–2.8)	1.1 (0.9–1.5)	< 0.001	84.8 (84.0-85.6)	15.2 (14.4–16.0)	< 0.001
Aware	21,863	87.9 (87.1–88.6)	10.5 (9.8–11.2)	1.1 (0.9–1.4)	0.5 (0.3–0.7)		82.1 (81.3–82.9)	17.9 (17.1–18.7)	

*It is based on the participant’s knowledge about the serious illness caused due to tobacco use. Considered aware if all of the questions were answered as ‘yes’ from H01 to H02

Table 2 depicts the socio-demographic variations in awareness levels. Overall, females who never consumed tobacco (Never-users) (64.7%) and dual users (64.7%) showed maximum awareness, followed by women exposed to SHS, SLT users, and smokers. Upon further disaggregation, middle-aged and married smokers, youngest and unmarried SLT users, dual users, and women exposed to SHS showed maximum awareness. Awareness increased with education and was high in urban areas (except in the cases of smokers, where women with more years of education and urban regions showed minimum knowledge). Minimum awareness was seen in smokers, SH smokers from central India, SLT users from North India, and dual users from South India. Women with positive intentions to quit had better knowledge, except for dual users.

**Table 2:** Awareness regarding adverse effects due to tobacco use among the female participants of the Global Adults Tobacco Survey (India)-round II (2016-17)

Characteristics	Awareness among									
	**Never users**		**Smokeless tobacco users**		**Smokers**		**dual tobacco users**		**Second-hand smoke-exposed**	
	Weighted % (95% CI)	p-value	Weighted % (95% CI)	p-value	Weighted % (95% CI)	p-value	Weighted % (95% CI)	p-value	Weighted % (95% CI)	p-value
Unweighted Numbers	**33,374**		**5559**		**824**		**508**		**6731**	
Overall	64.7(63.5–65.9)		43.0(36.7, 49.6)		50.6(48.0-53.1)		64.7(63.5–65.9)		54.8 (52.9–56.7)	
**Age group (years)**		< 0.001		< 0.001		< 0.001		< 0.001		< 0.001
15–29	53.9 (52.4–55.4)		24.3 (10.8–45.9)		44.7 (39.6–49.9)		27.2 (10.9–53.2)		57.5 (54.2–60.8)	
30–44	54.4 (53.0- 55.9)		38.3 (26.4–51.8)		43.3 (39.3–47.4)		33.7 (19.5–51.7)		55.7 (52.5–58.8)	
45– 59	52.3 (50.4–54.2)		38.5 (29.4–48.5)		39.3 (35.8–43.0)		45.5 (28.6–63.6)		51.9 (47.8–55.9)	
≥ 60	46.2 (43.7–48.7)		27.1 (20.5–34.9)		35.4 (31.3–39.7)		20.0 (10.3–35.3)		46.4 (40.7–52.3)	
**Marital status**		< 0.001		0.013		< 0.001		< 0.001		< 0.001
Unmarried	54.1 (43.5–48.3)		23.1 (4.3–66.9)		47.5 (36.6–58.6)		49.1 (9.1–90.3)		59.4 (54.1–64.4)	
Married	53.4 (52.4–54.4)		34.9 (28.7–41.7)		41.8 (39.4–44.3)		29.6 (19.6–42.0)		54.2 (52.1–56.3)	
Separated/divorced/widowed	47.0 (44.3–49.8)		28.2 (20.3–37.8)		34.4 (30.2–38.8)		30.2 (17.2–47.6)		49.5 (43.6–55.4)	
**Level of education**		< 0.001		< 0.001		< 0.001		< 0.001		< 0.001
No formal school	44.9 (43.5–46.5)		32.1 (26.9–37.7)		35.2 (32.7–37.9)		27.2 (18.4–38.1)		45.3 (42.1–48.4)	
Up to primary school	53.0 (51.0- 54.9)		30.1 (17.2–47.2)		47.1 (42.6–51.6)		38.5 (18.3–63.8)		52.9 (48.7–57.0)	
Up to secondary school	56.2 (54.6–57.8)		60.8 (29.6–85.1)		49.3 (43.1–55.5)		59.3 (28.4–84.3)		59.5 (55.7–63.1)	
Higher secondary and above	60.2 (58.2–62.2)		4.6 (0.8–22.3)		57.2 (42.0- 71.1)		43.1 (17.2–73.4)		64.2 (59.9–68.2)	
**Residence**		< 0.001		< 0.001		< 0.001		< 0.001		< 0.001
Urban	55.9 (54.4–57.4)		30.3 (18.8–44.9)		41.9 (37.0–47.0)		43.5 (22.7–66.8)		55.9 (52.6–59.1)	
Rural	51.3 (50.2–52.3)		32.9 (27.5–38.7)		39.6 (37.4–41.9)		28.3 (19.6–38.9)		54.1 (51.8–56.4)	
**Region**		< 0.001		< 0.001		< 0.001		< 0.001		< 0.001
North	55.6 (54.2–57.0)		44.6 (37.0- 52.5)		24.2 (16.3–34.4)		27.6 (13.1–49.1)		55.7 (52.9–58.6)	
Central	48.3 (46.3–50.3)		15.3 (10.0- 22.6)		39.1 (35.2–43.1)		23.6 (11.9–41.5)		46.6 (42.5–50.7)	
East	49.4 (47.5–51.2)		23.8 (15.1–35.4)		39.0 (35.2–42.9)		26.0 (11.5–48.7)		54.7 (50.4–58.9)	
North East	40.7 (38.1–43.4)		50.1 (37.5–62.7)		40.5 (37.4–43.7)		37.5 (29.2–46.6)		50.0 (45.1–54.9)	
West	61.0 (58.7–63.4)		54.8 (23.3–82.9)		50.9 (44.7–57.1)		53.6 (25.7–79.5)		64.1 (58.7–69.2)	
South	56.8 (55.2–58.5)		73.8 (57.3–85.6)		33.7 (28.4–39.3)		22.4 (9.9–43.3)		59.5 (55.5–63.3)	
**Wealth-index Quintiles**		< 0.001		< 0.001		< 0.001		< 0.001		< 0.001
First	57.3 (55.2–59.4)		57.7 (42.7–71.4)		46.6 (41.9–51.4)		20.4 (9.1–39.6)		57.8 (53.3–62.1)	
Second	52.6 (50.7–54.5)		37.5 (27.0- 49.4)		41.7 (37.5–45.9)		17.7 (7.8–35.2)		52.8 (48.6–56.9)	
Third	51.6 (49.7–53.4)		32.9 (22.9–44.6)		41.8 (37.6–46.3)		41.3 (22.7–62.7)		55.1 (50.5–59.6)	
Fourth	51.0 (49.0–53.0)		25.3 (17.2–35.6)		34.3 (29.6–39.3)		41.4 (24.8–60.1)		52.2 (48.1–56.2)	
Fifth	54.9 (52.9–56.8)		22.2 (13.0- 35.2)		35.1 (29.4–41.3)		23.5 (10.2–45.2)		58.2 (53.9–62.3)	
I**ntention to quit tobacco use**		< 0.001		< 0.001		< 0.001		< 0.001		< 0.001
Yes	NA		38.0 (30.0- 46.7)		43.9 (40.6–47.2)		30.0 (20.1–42.6)		43.5 (29.5–58.7)	
No	NA		25.4 (18.5–33.9)		37.9 (35.0–40.9)		36.2 (24.5–49.9)		25.5 (15.3–39.3)	

We then compared the effects of noticing the PHW on thoughts about quitting amongst the different types of tobacco users **(**Table 3**).** PHW was noticed more by the bidi smokers, followed by SLT users and cigarette smokers. PHW was noticed more in younger age groups, educated women from urban areas, and in the highest socio-economic quintile of society. The proportion of females who had thought about quitting tobacco usage because of PHW was highest amongst cigarettes, followed by bidi users, and was minimum for SLT users. Among cigarette smokers, thinking about quitting was most common in women between 45 and 59 years of age, from rural areas, with no formal school, and belonging to middle-class families. The highest proportion of bidi smoking women who thought about quitting was seen in the youngest age groups, educated up to primary school and belonging to the poorest quintile. Nearly half of the women (45.6%) thought about giving up SLT usage due to PHW. These women belonged to middle age groups, from urban areas, and were educated.

**Table 3:** Intention to quit tobacco among women who noticed a pack health warning on tobacco products as per the second round of the GATS-India.

Demographic characteristics	Current cigarette smokers (N = 697) who				Bidi Smoker (N = 420)				Smokeless tobacco (N = 5584)			
	**Noticed PHW**		**Thought about quitting** **because of PHW**		**Noticed PHW**		**Thought about quitting because of PHW**		**Noticed PHW**		**Thought about quitting because of PHW**	
	**Percentage** **(95% CI)**	**p-value**	**Percentage** **(95% CI)**	**p-value**	**Percentage** **(95% CI)**	**p-value**	**Percentage** **(95% CI)**	**p-value**	**Percentage** **(95% CI)**	**p-value**	**Percentage** **(95% CI)**	**p-value**
**Overall**	22.0 (17.1, 27.7)		**71.6 (58.1, 82.1)**		**54.4 (47.0, 61.5)**		52.1 (41.8, 62.3)		46.2 (43.1, 49.3)		45.6 (41.6, 49.6)	
**Age groups (**Years**)**												
15–29	**51.0 (18.7, 82.4)**	< 0.001	72.6 (21.3, 96.3)	< 0.001	**95.2 (81.5, 98.9)**	< 0.001	62.6 (11.2, 95.7)	< 0.001	63.4 (24.7, 90.1)	< 0.001	23.8 (0.4, 96.0)	< 0.001
30–44	30.9 (19.7, 44.9)		63.8 (35.2, 85.2)		63.2 (48.4, 75.8)		61.9 (42.0, 78.5)		**76.8 (58.1, 88.7)**		**55.7 (28.1, 80.2)**	
45–59	26.8 (18.2, 37.7)		**79.2 (60.2, 90.6)**		58.2 (45.2, 70.2)		49.2 (33.1, 65.5)		48.0 (24.1, 72.9)		52.8 (24.5, 79.4)	
≥ 60	11.6 (7.0, 18.6)		70.1 (46.7, 86.3)		45.5 (34.4, 57.1)		46.3 (29.7, 63.8)		43.3 (38.8, 47.9)		44.1 (36.8, 51.6)	
**Residence**												
Urban	**44.5 (28.8, 61.4)**	< 0.001	70.6 (45.5, 87.3)	< 0.001	**73.7 (53.0, 87.4)**	< 0.001	52.1 (27.8, 75.3)	0.326	**65.0 (60.0, 69.6)**	< 0.001	56.3 (49.1, 63.3)	< 0.001
Rural	19.5 (14.6, 25.5)		**71.9 (55.7, 83.9)**		52.3 (44.5, 60.0)		52.1 (40.9, 63.2)		51.6 (49.3, 53.9)		54.9 (51.5, 58.3)	
**Education**												
No formal schooling	15.6 (11.6, 20.6)	< 0.001	76.7 (63.9, 86.0)	< 0.001	51.4 (43.7, 58.9)	< 0.001	47.2 (36.7, 58.1)	< 0.001	48.8 (46.1, 51.5)	< 0.001	50.1 (46.1, 54.2)	< 0.001
Up to Primary education	60.6 (39.8, 78.1)		67.6 (33.9, 89.4)		81.2 (57.1, 93.4)		82.8 (44.9, 96.6)		58.7 (54.0, 63.2)		63.1 (56.8, 69.0)	
Up to secondary education	61.4 (29.0, 86.1)		50.8 (14.1, 86.7)		71.5 (18.7, 96.5)		58.5 (9.9, 94.8)		73.3 (67.7, 78.2)		57.8 (49.4, 65.8)	
Higher secondary and above	**80.7 (50.4, 94.5)**		57.3 (14.2, 91.6)		0		0		87.8 (52.1, 97.9)			
**Wealth index**												
First quintile	22.1 (12.5, 36.1)	< 0.001	74.7 (44.6, 91.5)	< 0.001	40.3 (21.7, 62.1)	< 0.001	73.9 (39.0, 92.6)	< 0.001	55.8 (51.0, 60.4)	< 0.001	53.7 (46.8, 60.4)	< 0.001
s quintile	16.3 (9.0, 27.8)		66.2 (37.8, 86.4)		61.7 (46.5, 74.9)		30.3 (16.4, 49.2)		56.0 (51.7, 60.2)		55.7 (49.7, 61.6)	
Third quintile	24.8 (14.5, 39.1)		78.2 (49.6, 92.9)		40.2 (26.1, 56.2)		57.3 (33.7, 78.0)		54.0 (49.4, 58.6)		52.7 (46.1, 59.2)	
Fourth quintile	23.7 (14.8, 35.8)		78.7 (58.6, 90.6)		54.5 (40.7, 67.7)		60.6 (39.0, 78.6)		53.0 (48.1, 57.9)		56.1 (48.2, 63.7)	
Fifth quintile	40.2 (19.8, 64.6)		45.1 (12.6, 82.4)		67.3 (42.6, 85.0)		58.3 (24.5, 85.8)		53.9 (47.4, 60.4)		64.1 (54.6, 72.5)	

Factors associated with improved awareness, observance of the PHW, and intentions to quit smoking for smokers and SLT users are shown in Tables 4 and 5. Multivariable binary logistic regression showed that the chances of having better awareness amongst the smoker women of middle ages (45–59 years), residing in urban areas, have received higher education, or belong to the poorest sections of society, were buying packed cigarettes, and believing that tobacco causes serious illness, while occupation, current pregnancy status, age of smoking initiation, the average number of cigarettes smoked per day, and time of first smoking upon waking up were observed to be non-significant in univariate logistic analysis and were not included in the final multivariable logistic model.

**Table 4:** Socio-demographic factors affecting the awareness levels, noticing the PHW, and intention to quit among the current smoker (female) who participated in the second round of GATS India

	Awareness		Noticing PHW		Intention to quit	
**Demographic characteristics**	**Unadjusted OR (95% CI)**	**Adjusted OR (95% CI)**	**Unadjusted OR (95% CI)**	**Adjusted OR (95% CI)**	**Unadjusted OR (95% CI)**	**Adjusted OR (95% CI)**
**Marital status**						
Unmarried	**Ref.**	**Ref.**	**Ref.**	**Ref.**	**Ref.**	**Ref.**
Married	0.8 (0.8–0.9)	0.4 (0.1–2.3)	0.4 (0.3–0.9)	0.6 (0.0-6.7)	0.34 (0.15–0.78)	0.2 (0.0-1.5)
Separated/divorced/widowed	0.6 (0.5–0.6)	0.2 (0.0-1.4)	0.3 (0.1–0.8)	0.2 (0.0-2.7)	0.27 (0.12–0.63)	0.1 (0.0-0.6)
**Age groups**						-
15–29 (Ref.)	**Ref.**	**Ref.**	**Ref.**	**Ref.**	**Ref.**	
30–44	1.2 (0.8–1.7)	3.1 (0.9–10.6)	1.1 (0.7–1.6)	1.1 (0.3–3.5)	1.4 (0.9–2.2)	-
45–59	0.9 (0.6–1.4)	11.5 (2.6–50.3)	1.0 (0.7–1.6)	1.3 (0.3–5.4)	1.2 (0.8–1.8)	-
≥ 60	0.6 (0.4–0.9)	4.1 (0.9–18.4)	0.6 (0.4–0.9)	1.4 (0.3–6.2)	0.8 (0.6–1.3)	-
**Residence**						-
Urban (Ref.)	**Ref.**	**Ref.**	**Ref.**		**Ref**	
Rural	0.5 (0.4–0.7)	0.8 (0.3–2.2)	0.4 (0.3–0.6)	0.6 (0.2–1.6)	0.7 (0.5-1.0)	-
**Education**						
No formal schooling	0.3 (0.1–0.8)	0.2 (0.0-0.8)	0.4 (0.1–0.9)	0.7 (0.2–2.6)	0.3 (0.1–0.7)	0.2 (0.0-1.1)
Up to Primary education	0.5 (0.23–1.3)	0.7 (0.2–3.1)	0.7 (0.3–1.7)	2.7 (0.5–13.2)	0.4 (0.1–0.9)	0.2 (0.0-1.2)
Up to secondary education	0.7 (0.3–1.7)	3.2 (0.7–15.1)	0.7 (0.3-2.0)	1.2 (0.2–7.7)	0.7 (0.3–1.9)	1.3 (0.2-8.0)
Higher secondary and above	**Ref.**	**Ref.**	**Ref.**	-	**Ref.**	**Ref.**
**Occupation**						
Govt./Non-Government employee	**Ref**	**-**	**Ref**	-	**Ref.**	**Ref.**
Self-Employed/Retired	1.1 (0.5–2.6)	-	1.0 (0.4–2.5)	-	0.8 (0.3–1.8)	1.5 (0.2–9.2)
Student/Homemaker	1.1 (0.5–2.3)	-	0.7 (0.3–1.5)	-	0.78 (0.36–1.7)	1.3 (0.2-7.0)
Unemployed	0.5 (0.2–1.2)	-	0.5 (0.2–1.2)	-	**0.3 (0.1–0.8)**	0.3 (0.0-3.1)
**Wealth index Quintiles**						
1	1.5 (1.0-2.4)	6.4 (1.2–34.9)	1.2 (0.7–1.8)	-	0.9 (0.6–1.4)	-
2	0.9 (0.6–1.4)	1.2 (0.2–5.5)	0.9 (0.6–1.3)	-	0.8 (0.5–1.1)	-
3	0.9 (0.6–1.3)	1.8 (0.5–7.8)	0.7 (0.5–1.1)	-	1.1 (0.7–1.6)	-
4	0.8 (0.5–1.2)	1.4 (0.5–4.2)	0.9 (0.7–1.4)	-	1.0 (0.7–1.5)	-
5	**Ref**	**Ref**	**Ref**		**Ref**	-
**Currently pregnant**						
Yes	**Ref**		**Ref**		**Ref**	
No	0.91 (0.82–1.02)		0.2 (0.0-1.8)	-	0.3 (0.1–1.5)	-
**Age at daily Smoking initiation**						-
Less than 15 (Ref.)	**Ref**	-	**Ref**	-	**Ref**	-
15–18 years	0.8 (0.5–1.4)	-	1.4 (0.9–2.4)	-	1.2 (0.7-2.0)	-
19–21 years	1.2 (0.8–1.9)	-	1.3 (0.8–2.1)	-	1.2 (0.8–1.9)	-
22 years old or older	0.8 (0.5–1.1)	-	1.1 (0.7–1.5)	-	1.4 (0.9-2.0)	-
**The average number of cigarettes smoked per day.**						-
Less than 5 Cigs/day	**Ref.**	-	**Ref.**	-	**Ref.**	
5–9 Cigs/day	0.9 (0.6–1.3)	-	1.9 (1.3–2.8)	-	0.8 (0.6–1.1)	-
10–14 Cigs/day	0.6 (0.4-1.0)	-	1.8 (1.1-3.0)	-	0.9 (0.5–1.4)	-
15–24 Cigs/day	1.0 (0.6–1.9)	-	1.8 (0.9–3.5)	-	0.8 (0.4–1.4)	-
>=24 Cigs/day	0.9 (0.5–1.8)	-	2.5 (1.2-5.0)	-	0.5 (0.3-1.0)	-
**How do you buy cigarettes?**						
Loose cigarettes	**Ref**	**Ref**	**Ref**	-	**Ref**	**Ref**
Packets	0.4 (0.2–0.9)	1.17 (1.10–2.96)	0.4 (0.2–0.9)	2.21 (1.48–3.66)	0.4 (0.2–0.7)	1.3 (1.0-3.5)
**Believes that tobacco causes Serious Illness**						-
No	**-**	**-**	**Ref**	**Ref**	**Ref**	**Ref**
Yes	-	-	3.6 (2.5-5.0)	7.35 (2.58–20.91)	2.8 (2.0-4.1)	6.7 (2.1–21.6)
**Time of First Smoking Upon Waking**						
Within 5 min	**Ref**	-	**Ref**	-	**Ref**	-
Within 6–30 min	1.0 (0.7–1.4)	-	1.2 (0.8–1.7)	-	**0.6 (0.4–0.9)**	-
Within 31–60 min	1.0 (0.6–1.5)	-	1.5 (0.9–2.3)	-	1.0 (0.7–1.6)	-
More than 60 min	0.8 (0.5–1.2)	-	1.0 (0.7–1.5)	-	1.4 (1.0-2.2)	-
**Smoking Quit Attempt in the Past 12 Months**						
No	**Ref**	-	**Ref**	-	**-**	**-**
Yes	1.0 (0.7–1.3)	-	**1.5 (1.1–1.9)**	**1.9 (1.1–5.3)**	**-**	**-**

**Table 5:** Socio-demographic factors affecting the knowledge, noticing PHW, and intention to quit among the current Smokeless tobacco users (Females)who participated in the second round of GATS India

	Knowledge		Noticing PHW		Intention to quit	
	**Unadjusted OR (95% CI)**	**Adjusted OR (95% CI)**	**Unadjusted OR (95% CI)**	**Adjusted OR (95% CI)**	**Unadjusted OR (95% CI)**	**Adjusted OR (95% CI)**
**Marital status**						
Unmarried	**Ref**	**Ref**	**Ref**	**Ref**	**Ref**	**Ref**
Married	0.4 (0.4–0.6)	0.7 (0.5–1.1)	0.6 (0.5–0.8)	1.2 (0.8–1.7)	0.4 (0.1–1.2)	1.2 (0.2-6.0)
Separated/divorced/widowed	0.3 (0.2–0.4)	0.6 (0.4-1.0)	0.3 (0.2–0.4)	0.8 (0.5–1.2)	0.3 (0.1–0.9)	0.8 (0.1–4.5)
**Age groups (years)**						
15–29	**Ref**	**Ref**	**Ref**	**Ref**	**Ref**	**Ref**
30–44	0.9 (0.7-1.0)	1.1 (0.9–1.4)	1.0 (0.8–1.2)	1.0 (0.8–1.3)	0.9 (0.7-1.0)	1.0 (0.4–2.8)
45–59	0.7 (0.6–0.9)	1.0 (0.8–1.3)	1.1 (0.9–1.4)	0.9 (0.7–1.2)	0.7 (0.6–0.8)	1.3 (0.4–3.6)
≥ 60	0.4 (0.4–0.5)	1.0 (0.7–1.3)	1.9 (1.5–2.3)	0.7 (0.5–0.9)	0.4 (0.3–0.4)	0.9 (0.3–2.8)
**Residence**						
Urban	**Ref**	**Ref**	**Ref**	**Ref**	**Ref**	**Ref**
Rural	0.5 (0.5–0.6)	0.7 (0.6–0.9)	0.5 (0.6 − 0.4)	0.6 (0.5–0.7)	0.6 (0.6–0.7)	0.6 (0.3–1.3)
**Education**						
No formal schooling	0.2 (0.1–0.3)	0.3 (0.2–0.5)	3.1 (2.2–4.4)	0.3 (0.2–0.4)	0.3 (0.2–0.3)	0.0 (0.0-0.5)
Up to Primary education	0.3 (0.2–0.5)	0.6 (0.4–0.9)	1.7 (1.2–2.4)	0.6 (0.4–0.9)	0.4 (0.3–0.56)	0.1 (0.01–1.7)
Up to secondary education	0.6 (0.4–0.9)	0.8 (0.5–1.1)	1.2 (0.8–1.7)	0.7 (0.4-1.0)	0.7 (0.5–0.9)	0.3 (0.0-3.3)
Higher secondary and above	**Ref**	**Ref**	**Ref**	**Ref**	**Ref**	**Ref**
**Occupation**						
Govt./Non-Govt. employee	**Ref**	**Ref**	**Ref**	**Ref**	**Ref**	
Self-Employed/Retired	0.8 (0.6–1.1)	0.8 (0.6–1.1)	1.5 (1.0-2.3)	1.5 (1.0-2.2)	0.6 (0.5–0.8)	-
Student/Homemaker	0.7 (0.5-1.0)	0.9 (0.7–1.3)	2.4 (1.7–3.4)	0.9 (0.7–1.4)	0.7 (0.5–0.9)	-
Unemployed	0.4 (0.3–0.6)	0.8 (0.5–1.2)	2.4 (1.6–3.8)	0.9 (0.5–1.4)	0.5 (0.3–0.7)	-
**Wealth index**						
1	1.0 (0.8–1.2)	0.8 (0.6-1.0)	0.5 (0.4–0.7)	0.8 (0.6–0.9)	0.5 (0.5–0.7)	2.3 (0.9-6.0)
2	0.8 (0.7-1.0)	1.0 (0.8–1.3)	0.9 (0.7–1.1)	0.9 (0.7–1.1)	0.6 (0.5–0.8)	2.7 (1.1–6.8)
3	0.9 (0.8–1.1)	0.6 (0.5–0.8)	1.0 (0.8–1.2)	0.7 (0.5–0.8)	0.7 (0.5–0.8)	2.2 (0.9–5.5)
4	0.7 (0.6–0.8)	0.9 (0.7–1.1)	1.3 (1.1–1.6)	0.7 (0.5–0.8)	0.7 (0.6–0.9)	1.8 (0.6–5.5)
5	**Ref**	**Ref**	**Ref**	**Ref**	**Ref**	**Ref**
**Currently pregnant**						
Yes	**Ref**		**Ref**		**Ref**	
No	0.9 (0.7–1.3)	-	0.7 (0.5–1.1)	-	0.2 (0.0–1.5)	-
**Age at daily Smoking initiation**						
Less than 15	**Ref**	**Ref**	**Ref**	**Ref**	**Ref**	**Ref**
15–18 years	1.5 (1.2–1.9)	1.2 (1.0-1.6)	0.8 (0.7–1.1)	0.8 (0.6–1.1)	1.7 (1.4–2.1)	0.9 (0.3–2.5)
19–21 years	1.1 (0.9–1.3)	1.1 (0.9–1.4)	0.7 (0.6–0.9)	1.0 (0.8–1.3)	1.4 (1.2–1.7)	0.6 (0.2–1.5)
22 years old or older	1.2 (1.0-1.5)	0.9 (0.8–1.2)	0.7 (0.6–0.8)	1.0 (0.8–1.2)	1.5 (1.3–1.8)	0.8 (0.4-2.0)
**Type of smokeless tobacco**						
Single Use Pouch	**Ref**	**Ref**	**Ref**	**Ref**	**Ref**	**Ref**
Large pouch/Can	1.0 (0.8–1.2)	0.7 (0.59–0.9)	0.7 (0.5–0.8)	1.4 (1.1–1.7)	0.7 (0.6–0.9)	0.4 (0.1–1.1)
Loose product	0.5 (0.4–0.6)	0.6 (0.5–0.7)	1.2 (1.7–1.4)	0.8 (0.6–0.9)	0.9 (0.8–1.1)	1.0 (0.5–1.9)
**Use smokeless tobacco after waking.**						
Within 5 min	**Ref**	**Ref**	**Ref**	**Ref**	**Ref**	**Ref**
6 to 30 min	1.5 (1.2–1.8)	1.2 (1.0-1.5)	1.4 (1.2–1.8)	0.9 (0.7–1.2)	1.3 (1.1–1.6)	0.7 (0.3–1.5)
31 to 60 min	1.6 (1.3-2.0)	1.3 (1.0-1.6)	0.8 (0.7–1.1)	1.3 (1.0-1.7)	1.4 (1.1–1.7)	2.1 (0.8–5.7)
More than 60 min	1.2 (1.0-1.4)	1.1 (0.9–1.4)	0.7 (0.6–0.9)	1.5 (1.2–1.9)	1.9 (1.5–2.2)	1.7 (0.7–4.3)

Univariate analysis showed the effect of middle age, residential status, better education, average smoking per day, time of first smoking upon waking up, buying packed cigarettes/beedi, believing that tobacco causes serious illness, and making smoking quit attempts in the last 12 months on increased chances of noticing PHW. However, multivariable analysis showed only the effect of the previous three variables described above. There were higher odds of noticing PHW in unmarried smoker women, who were > 60 years of age, educated up to primary school, from urban areas, preferred to buy packed cigarette/bidi, believed that smoking causes serious illness, and those who attempted to quit smoking in last 12 months. Factors that positively affected intention to quit smoking included younger age, secondary school education, self-employed status, buying packed cigarettes/bidi, believing that smoking causes serious illness, and attempted quitting in the last 12 months.

Similarly, factors affecting awareness, noticing the PHW, and intention to quit among SLT users were assessed **(**Table 5**)**. The odds of having better awareness and noticing the PHW among SLT users were better for unmarried females, urban residents with more years of education, higher socio-economic status, single-use pouch, and lesser addiction. However, the intention to quit SLT was affected by only more years of education and lower-middle-class status.

## Discussion

The tobacco epidemic is swiftly claiming the lives of women and children. Though women’s tobacco use has declined in India, according to the GATS-2 data, the difference between men’s and women’s usage rates has remained nearly unchanged. Women bear a significantly more significant burden of tobacco-related disease and mortality. The tobacco industry has been at the forefront of the tobacco epidemic. In response to the substantial decline in tobacco consumption in Western countries over the last two decades, the tobacco industry has responded by focusing on women in LMIC as new potential customers. [27–29].

Our study found a high prevalence of SLT, SHS, and dual tobacco use among women. This load appears low when comparing male and female genders due to the former’s significantly larger consumption. As per the GATS atlas, 2015 tobacco use among Indian women as per round 1 (2010) was estimated to be 20%, placing them seventh out of 22 countries surveyed by GATS [30]. The prevalence decreased further as per the second round of GATS to 14.2% [31]. There is an overwhelming usage of SLT by Indian women (12.3%). In India, dual tobacco usage at 0.5% among women is high (or 5.3% of all current female tobacco users aged 15 and above) compared to other south-east Asian countries like Bangladesh, Malaysia, Thailand, and Indonesia [30, 31]. Because of under-reporting or the dearth of reliable data, low rates of female tobacco usage in Asian and African countries are likely higher than estimated [32, 33]. For instance, it was recently reported that the rate of cotinine-verified smokers in Korea was 8% higher than the rate of self-reported smokers [32].

We observed that the prevalence of ever-usage varied significantly with the socio-demographic characteristics like age of women, marital status, education, residential status, wealth quintiles, etc. Tobacco usage initiation in young women has been promoted by tobacco marketing, and some of the first advertisements posed cigarettes as a means of weight loss [34]. Currently, markets are inundated with advertisements that associate tobacco use with social desirability and women’s empowerment. It’s worth noting that, while men adopt smoking for the euphoric effects of nicotine, women smoke to experience just the smoke-related stimuli [35].

In our study, SLT usage was higher amongst the poorest quintiles, while smoking and dual-use were more common in affluent sections. This is incoherence to a multi-country analysis as per which, 90% of SLT burden is concentrated in LMICs, specifically among the poorest ones [36]. This is because the burden of SLT has not received adequate attention on a global scale. Taxation on SLT products is typically lower compared to cigarettes. This has eventually increased the acceptability of SLT due to enhanced affordability, and consumption dynamics are shifting from smoked to SLT forms [37, 38]. evidence suggests that implementing increased taxation on raw tobacco and SLT products is an apposite tool for reducing SLT use by striking the affordability component of the user behavior [39]. Though Indian women prefer smoking less, there is substantial evidence of a high propensity for SLT use [40]. Previous research has linked SLT used to poor oral health and perinatal morbidities, such as premature birth, low birth weight, and birth length [41–43]. Also, these consequences are dose-responsive [44]. Indian women generally support using SLT to improve oral health and as a treatment for gastric problems, apart from enhancing companionship through shared use and a stress remedy [45]. Poor women working as laborers consume SLT to increase energy for heavy work and suppress hunger [42]. On the other hand, pregnant women begin using SLT because of a myth that chewing tobacco can help maintain the teeth and gums strength during pregnancy [45].

We observed that age, education, and wealth status impacted women’s awareness of the harmful effects of tobacco. This is consistent with previous evidence, which shows that education and income are related to knowledge about the detrimental effects of tobacco on women’s health [46–49]. There is an unending list of harmful effects of tobacco usage on women. The risk of developing COPD and its variants like chronic bronchitis or emphysema, consequently premature death, is higher in women smokers (approximately 22 times more than non-smokers) [50]. They are more likely to develop cancers of the oral cavity, esophagus, pancreas, kidney, bladder, and uterine cervix. They also have a twofold higher risk of developing coronary heart disease. Postmenopausal women smokers have decreased bone mineral density and higher chances of hip fracture, unlike non-smokers women [51]. Smoking also causes premature aging due to skin wrinkling [52].

We observed that the PHW labels were noticed maximum on Bidi packings, followed by SLT and cigarettes and that too in younger literate females from urban areas. However, there were higher odds of noticing if the women bought packed tobacco or had strong knowledge regarding the harmful effects of tobacco. According to previous studies in India, only a small percentage of cigarette packs and an even lower proportion of SLT products represented compliant PHWs [53]. . This initial bottleneck is particularly problematic in delivering the desired effect of PHW amongst the users from different areas of the country and with varied socio-demographic characteristics. However, women perceive PHW as more aversive than men and smokers, and women with lower education perceive them as more aversive than non-smokers and respondents with higher education [54]. There is strong evidence from previous analysis that supports the effectiveness of the PHW in influencing quit intentions, increased concerns about the adverse effects, and adoption of the sedation behavior [54].

We observed that Quit attempts were significantly affected by the PHW. According to GATS, cross-country variations in women’s quitting intentions can range between 33.8–82.8% [30]. A study of stress responses and cravings among male and female smokers attempting to quit discovered a lower level of cortisol - a stress hormone- responsible for the relapse in men during abstinence [55]. On the contrary, women’s cortisol levels increase and favor relapse [56]. Other research discovered that smoking alternative forms of nicotine cigarettes during abstinence could exacerbate the withdrawal symptoms and have more pronounced mood effects in men than in women. Women experience a similar level of stimuli from cigarettes that may or may not have nicotine, implying a less substantial role of the chemical as a predictor of smoking than men [57]. Previous studies that have analyzed quit attempts observed that women were 30% less likely to have successful quit attempts [58]. This was explained by the concerns of possible weight gain in the post-cessation period, which may not be accurate for women with less education and low awareness about the effect of tobacco on women’s weight [59]. However, a medical practitioner’s advice play a significant role in successful cessation [60]. Therefore, it is pertinent for doctors to counsel their female clients about the adverse effects of tobacco usage regardless of their specialty.

To conclude, it should be stressed that to achieve the UN target of a 30% reduction in tobacco use by 2025, greater attention to the burden of SLT use is required, particularly in LMICs, to implement evidence-based tobacco control strategies [61]. Tobacco regulators are particularly concerned about women because they are a current target of tobacco advertisements and promotion. The interventions on raising awareness and helping women tobacco users quit must be strategized according to the socio-demographic stratum, given the sociocultural diversification of India. Besides, a strict monitoring mechanism of OTT platforms and social media must be in place to check surrogate advertisements and glamorization of tobacco use. Mobilization of self-help groups, civil society advocates, and organizations working for women and children could assist and support broader campaigns to generate awareness and motivate users to quit. The tobacco control policies and intervention services for hard-to-reach areas and particular subgroups among women and youth should cater to their socio-demographic characteristics to pitch broader dissemination. The involvement of more female and youth ambassadors in tobacco control could set in a real-time advocacy mechanism to steer the tobacco-free movement in the country. In addition, there is a pressing need to implement an institutional mechanism rooted in systems thinking approach to support and achieve Sustainable Development Goals.

## Data Availability

The dataset is available at Global Tobacco Surveillance System (GTSS), Centers for Disease Control and Prevention (CDC), Global Adult Tobacco Survey- 2(2016–2017), India. (https://nccd.cdc.gov/GTSSDataSurveyResources/Ancillary/DataReports.aspx?CAID=2) and data were retrieved using standard protocols.

## Abbreviations

LMIC: Low Middle-Income Countries
WHO: World Health Organization
GATS: Global Adult Tobacco Survey
NFHS: National Family Health Survey
SLT: Smokeless Tobacco
FCTC: Framework Convention on Tobacco Control
MoHFW: Ministry of Health and Family Welfare
PHW: Pack Health Warning
SHS: Second Hand Smoke
